# Nephroblastoma in a Sprague Dawley rat unrelated to titanium dioxide nanoparticle exposure *in utero*

**DOI:** 10.1002/vms3.405

**Published:** 2020-12-05

**Authors:** Samantha J. Glaspell, Katie J. Knapek, Ida M. Washington, Scott D. Fitzgerald, Jessica S. Fortin

**Affiliations:** ^1^ Office of Laboratory Animal Resources West Virginia University Morgantown WV USA; ^2^ Department of Pathobiology and Diagnostic Investigation College of Veterinary Medicine Michigan State University East Lansing MI USA; ^3^ Veterinary Diagnostic Laboratory Michigan State University Lansing MI USA

**Keywords:** metastasis, nephroblastoma, rodent, TiO_2_, Wilms' tumour

## Abstract

Nephroblastoma is an embryonal tumour that has rarely been reported in laboratory rats. In this case report, a large nephroblastoma with peritoneal seeding was found during necropsy in an 11‐month‐old, female, Sprague Dawley rat. The rat had a history of indirect exposure to nano‐TiO_2_ (titanium dioxide nanoparticles) during maternal gestation. A firm mass in the upper right abdominal quadrant was palpated. Four weeks later, the animal quickly declined. Nephroblastoma was confirmed by histopathology. Only one rat developed nephroblastoma among the ten littermates. Nephroblastomas in Sprague Dawley rats are typically spontaneous tumours with non‐malignant mesenchymal elements. The capability to induce a nephroblastoma with nano‐TiO_2_ is less likely in this case.

## Graphical abstract

Nephroblastoma with serosal seeding masses in an 11‐month‐old, female, Sprague Dawley rat.

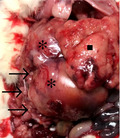

## 1 INTRODUCTION

In human medicine, nephroblastoma, commonly called Wilms' tumour, is the most common renal neoplasm of children. Typically, these tumours are made up of stromal, epithelial, or blastemal cells and affect one or both kidneys (Jackson & Kirkpatrick, 2002; Yoshizawa et al., 2013). These tumours have also been described commonly in pigs, chickens, dogs, cats and to a lesser extent bovine fetuses (Hayashi et al., 1986; Kirkbride & Bicknell, 1972; Montinaro et al., 2013; Potkay & Garman, 1969; Tsukamoto et al., 1998). In domestic animals, this tumour has a poor prognosis even with therapeutic intervention, and metastasis has been known to occur to the lymphatic system, lungs and liver (Montinaro et al., 2013). In laboratory mice, rats and rabbits, the occurrence of nephroblastoma is rare (Katsuta et al., 2017; Mesfin & Breech, 1992). Nephroblastomas often develop during foetal life and are not detected until the animal becomes symptomatic, usually later in life (Mesfin & Breech, 1992). In laboratory rats, this tumour can occur spontaneously or it can be chemically induced with agents such as N‐nitrosodimethylamine or N‐Methyl‐N‐nitrosourea (Hard & Grasso, 1976; Yoshizawa et al., 2013). Morphologically, the chemically induced nephroblastomas in rats closely resemble those observed in humans (Bannasch & Zerban, 1994; Yoshizawa et al., 2013).

## 2 CASE HISTORY

An 11‐month‐old female, Sprague Dawley rat (Hilltop Laboratories), presented with a 4‐week history of progressive abdominal distention. The rat was pair housed in an individually ventilated cage (IVC) on a 12:12 reverse‐light cycle with free access to water. This rat was indirectly exposed to titanium dioxide nanoparticles (Aeroxide®_TiO_2_ P 25 from Evonik Industries) during maternal gestation. Initially, the rat was bright, alert and responsive with a firm mass palpated in the upper right quadrant of the abdomen. The animal had no change in behaviour, and no other abnormalities were noted on the physical exam. The principal investigator declined diagnostic imaging of the rat and elected to monitor clinical signs. Four weeks after the initial exam, the rat exhibited discomfort, hypothermia and extreme lethargy. The abdomen was easily palpated and a large firm mass with irregular edges was palpable in the upper right quadrant of the abdomen. It was difficult to determine if the mass was a tumour or fetus. Despite daily staff monitoring and supportive care, the rat's condition continued to decline. The senior laboratory member elected to euthanize the rat and have the veterinary team conduct a necropsy.

## 3 MATERIAL AND METHODS

### 3.1 Postmortem examination

All animals were used in accordance with protocols approved in advance by the Institutional Animal Care and Use Committee at their respective facilities. Postmortem examination was performed on the Sprague Dawley rat with the abdominal mass. Tissues were formalin‐fixed and stained with haematoxylin and eosin. Two board‐certified pathologists examined the masses for diagnostic confirmation. The rat was indirectly exposed to nano‐TiO_2_ (titanium dioxide nanoparticles) during maternal gestation. The experimental protocol of the inhalation study is detailed for completeness of case history.

### 3.2 Nanomaterial aerosol characterization

Nano‐TiO_2_ P25 powder was obtained from Evonik (Aeroxide TiO_2_). This powder is a mixture containing anatase (80%) and rutile (20%) TiO_2_, with a primary particle size of 21 nm, a surface area of 48.08 m^2^/g, and a Zeta potential of −56.6 mV (Stapleton et al., 2018). The TiO_2_ powder was stored in a glass desiccator to maintain dryness for aerosolizing. Aerosol size distributions were measured during aerosol exposures while TiO_2_ mass concentration was maintained at the target concentration of approximately 12 mg/m^3^ using: (a) a high‐resolution electrical low‐pressure impactor (ELPI+; Dekati), (b) a scanning mobility particle sizer (SMPS 3938; TSI, Inc.), (c) an aerodynamic particle sizer (APS 3321; TSI Inc.) and (d) a nano micro‐orifice uniform deposit impactor (MOUDI 115R, MSP Corp). This 12 mg/m^3^ concentration is below the permissible exposure limit set by the Occupational Safety and Health Administration (OSHA; 15 mg/m^3^; CDC‐NIOSH, 2011).

### 3.3 Whole‐body inhalation exposure

Inhalation exposures were conducted using a high‐pressure acoustical generator (HPAG, IEStechno) to produce nano‐TiO_2_ aerosols as previously described (Abukabda et al., 2019). After leaving the acoustical generator, the aerosols were fed through a Venturi pump (JS‐60 M, Vaccon) to further deagglomerate the particles prior to entering the exposure chamber. A personal DataRAM (pDR‐1500; Thermo Fisher Environmental Instruments, Inc.) was used to sample the exposure chamber air in real‐time, and a software controller automatically adjusted the acoustic energy to maintain a constant aerosol mass concentration (12 mg/m^3^) during exposures. Gravimetric measurements were conducted using Teflon filters in the breathing zone of the animals (sample flow = 0.35 L/min) to quantify the average aerosol mass concentrations for each day of exposure. Sham controls were exposed to HEPA‐filtered air, but all other chamber conditions (i.e. temperature, humidity) were held constant.

Four pregnant adult female Sprague Dawley rats (Hilltop Laboratories) were used to produce litters following inhalation exposures (two nano‐TiO_2_; two air control). Rats were housed in AAALAC‐approved facilities with ad libitum access to food and water. Female rats were monitored for oestrus, at which time each female rat was placed with an individual male rat. Female rats subsequently were given a vaginal smear every 12 hr to verify mating via the presence of sperm. Exposures began on GD 11 to allow for uterine implantation of the embryo (Stapleton et al., 2013). Pregnant rats were exposed to a target concentration of 12 mg/m^3^ for 6h/day for 6 days, which was chosen based on previous studies showing compromised cardiovascular health and behavioural deficits in rats following similar exposures (Engler‐Chiurazzi et al., 2016; Stapleton et al., 2013, 2018). Daily total lung dose of nano‐TiO_2_ aerosols was estimated by accounting for the mass proportion of particles deposited in the rat lung (10%), the average volume of air breathed in by a rat per minute (208.33 cc), the TiO_2_ mass concentration (12 mg/m^3^), and the exposure duration in minutes (360 min; Yi et al., 2013). The cumulative lung dose of the exposure paradigm (6 hr/day, 6 days) was estimated to be approximately 525 µg (Abukabda et al., 2019). The final exposure occurred approximately 48 hr prior to pup delivery.

### 3.4 Subjects

Twenty‐nine adult male and female Sprague Dawley rats (Male = 15 [nano‐TiO_2_ = 7; control = 8]; Female = 14 [nano‐TiO_2_ = 6; control = 8]) across the four litters (two nano‐TiO_2_; two sham control) were used as subjects. After weaning at PND 21, rats were pair‐housed within exposure groups and sex in a temperature‐ and humidity‐controlled (72°F, 60%) vivarium operating on a reverse 12:12 hr dark/light cycle. Until PND 60, food and water were freely available in the home cage. Starting on PND 60, unless otherwise specified, food was restricted to 12–15 g of rat chow per rat per day with water freely available. Rats received this daily allotment of food 30 min after daily testing resulting in approximately 22 hr of food restriction prior to sessions. Experimental sessions were conducted five or six days per week at approximately the same time each day during the rats' dark cycle. Additionally, sessions for male and female rats were always conducted in separate operant‐conditioning chambers. Rats were maintained in accordance with National Institutes of Health guidelines for Care and Use of Laboratory Animals, and the West Virginia University Animal Care and Use Committee approved all experimental procedures.

### 3.5 Differential‐reinforcement‐of‐low‐rates (DRL)

Rats began DRL training 80 days following completion of the final reversal at approximately 6 months of age due to restriction of laboratory activities outside the experimenter's control. During this 80‐day break, all rats remained in the colony room with free access to water and the same daily ration of food. Rats were initially trained on a DRL 5‐s schedule. On this schedule, only interresponse times (IRTs) ≥5 s were reinforced. Thereafter, the DRL schedule value increased across a preset number of sessions to reach the terminal 20 and 30‐s schedules. The DRL schedule progression was as follows: 10 sessions of DRL 5 s, 15 sessions of DRL 10 s, 15 sessions of DRL 20 s and 20 sessions of DRL 30 s. Rats could earn up to 60 pellets per session, and session duration was set to 60 min.

## 4 RESULTS

**FIGURE 1:**
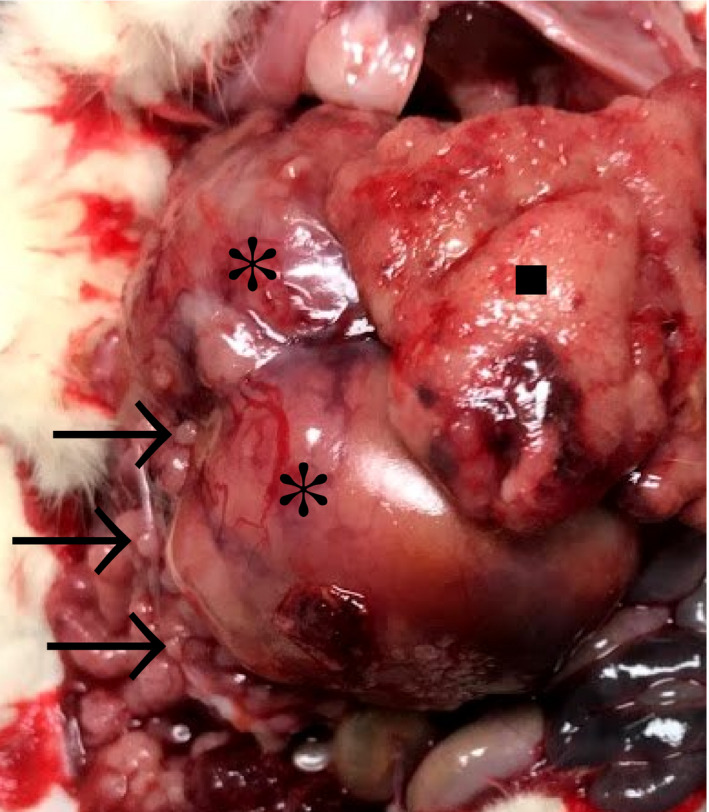
Macroscopic picture of the rat abdominal mass. A large expansive abdominal bi‐lobed mass (starbursts) was associated with multifocal to coalescing, 5‐50 mm, tan nodules (arrows). The liver was pale (square)

At necropsy, a large highly vascularized, bi‐lobed, spherical mass, with irregular edges filled the majority of the abdomen (Figure 1). The mass extended caudally from the left kidney. There were too‐numerous‐to‐count tan nodules of 2–5 mm diameter on the omental, serosal surface of the reproductive tract and the primary mass. Representative samples of the lungs, pancreas, heart, liver, mesentery, intestinal serosa and the abdominal mass were collected and placed in 10% neutral‐buffered formalin.

**FIGURE 2:**
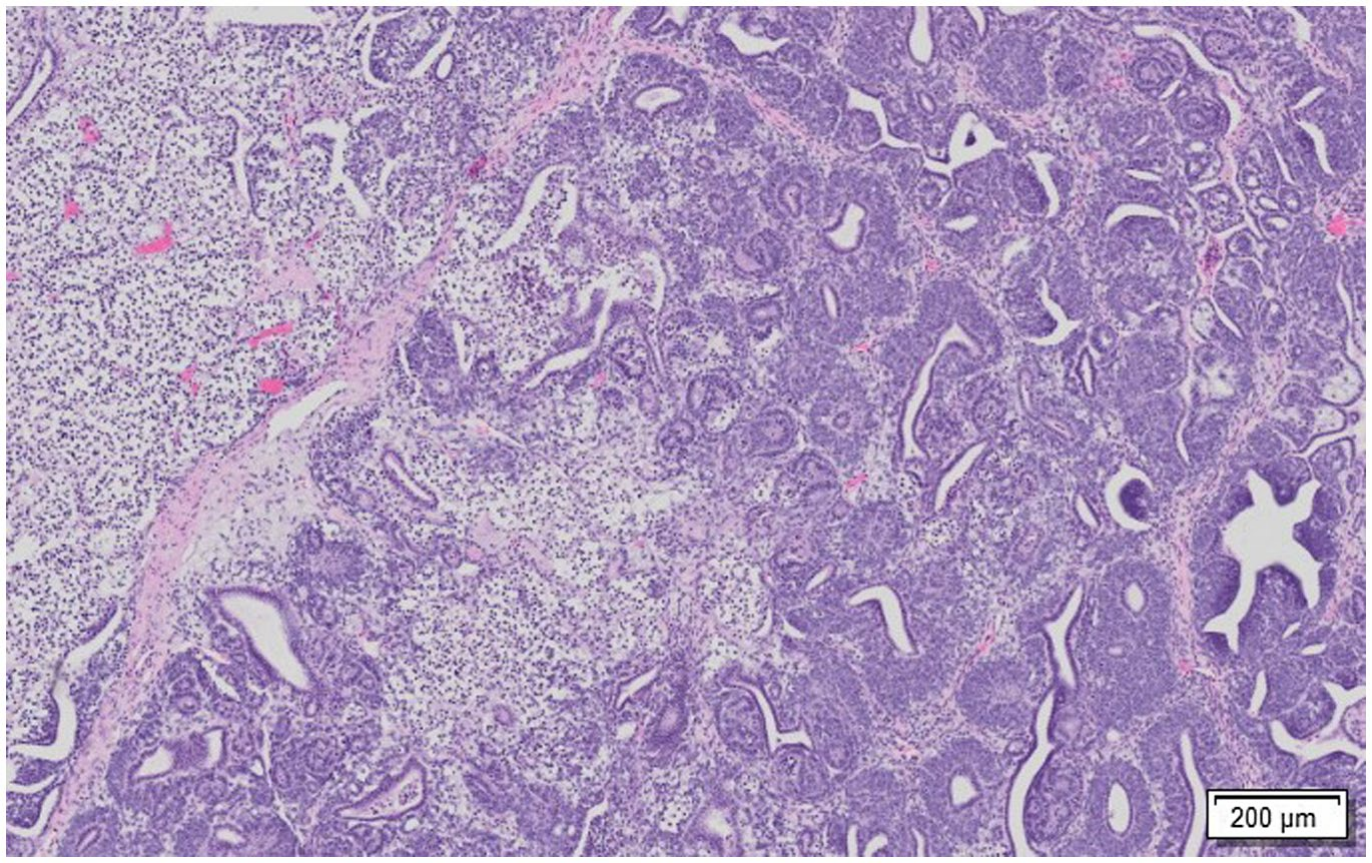
Photomicrograph of sections of the affected kidney (haematoxylin and eosin [H&E] stain). Histologically, the large abdominal mass observed during gross examination exhibited a biphasic pattern comprised of epithelial and mesenchymal elements

**FIGURE 3:**
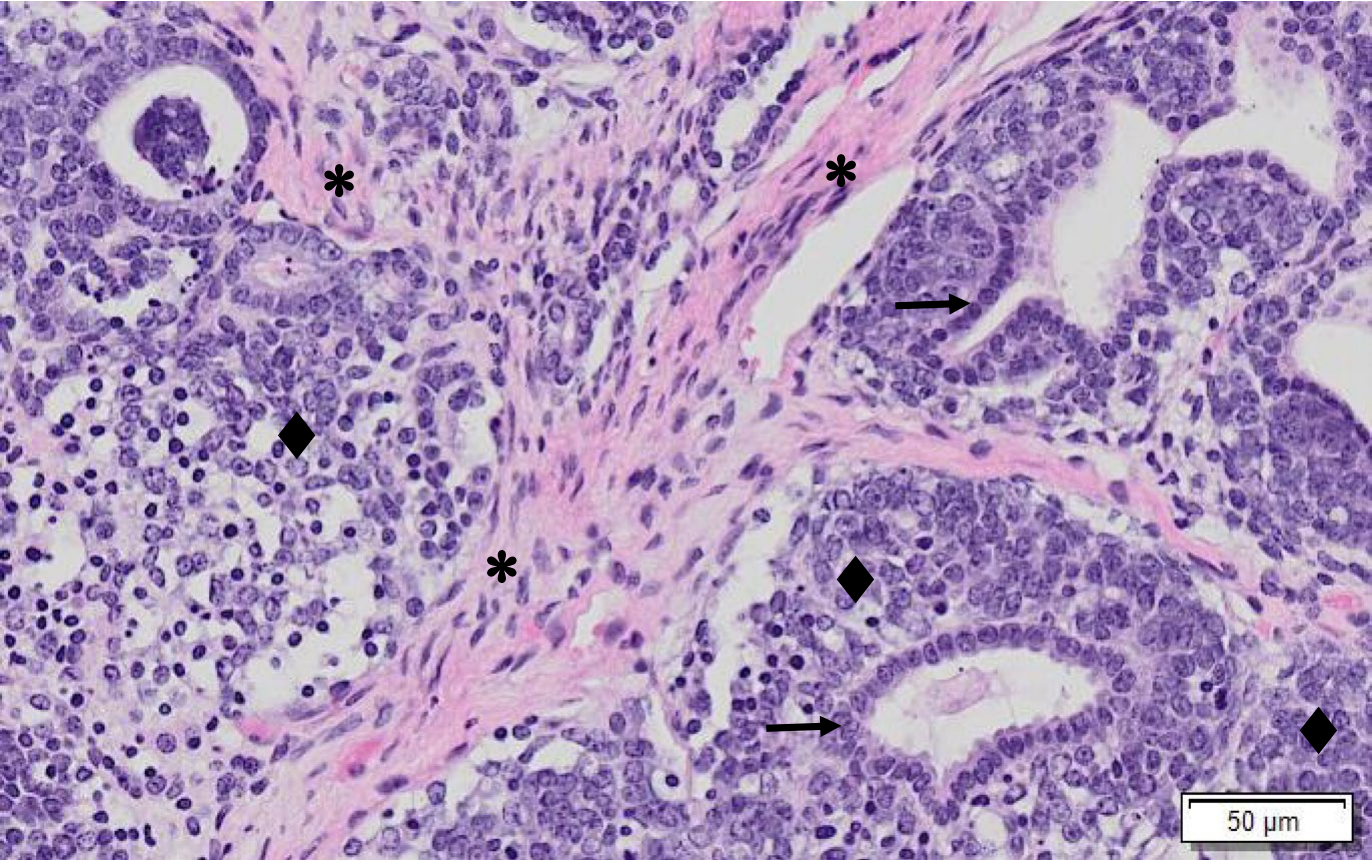
Higher magnification of the photomicrograph of tissue section of renal mass (haematoxylin and eosin [H&E] stain) to show the epithelial components (lozenge) with tubular structures (arrows) and spindle cells (mesenchymal component) between the tubule profiles (starburst)

**FIGURE 4:**
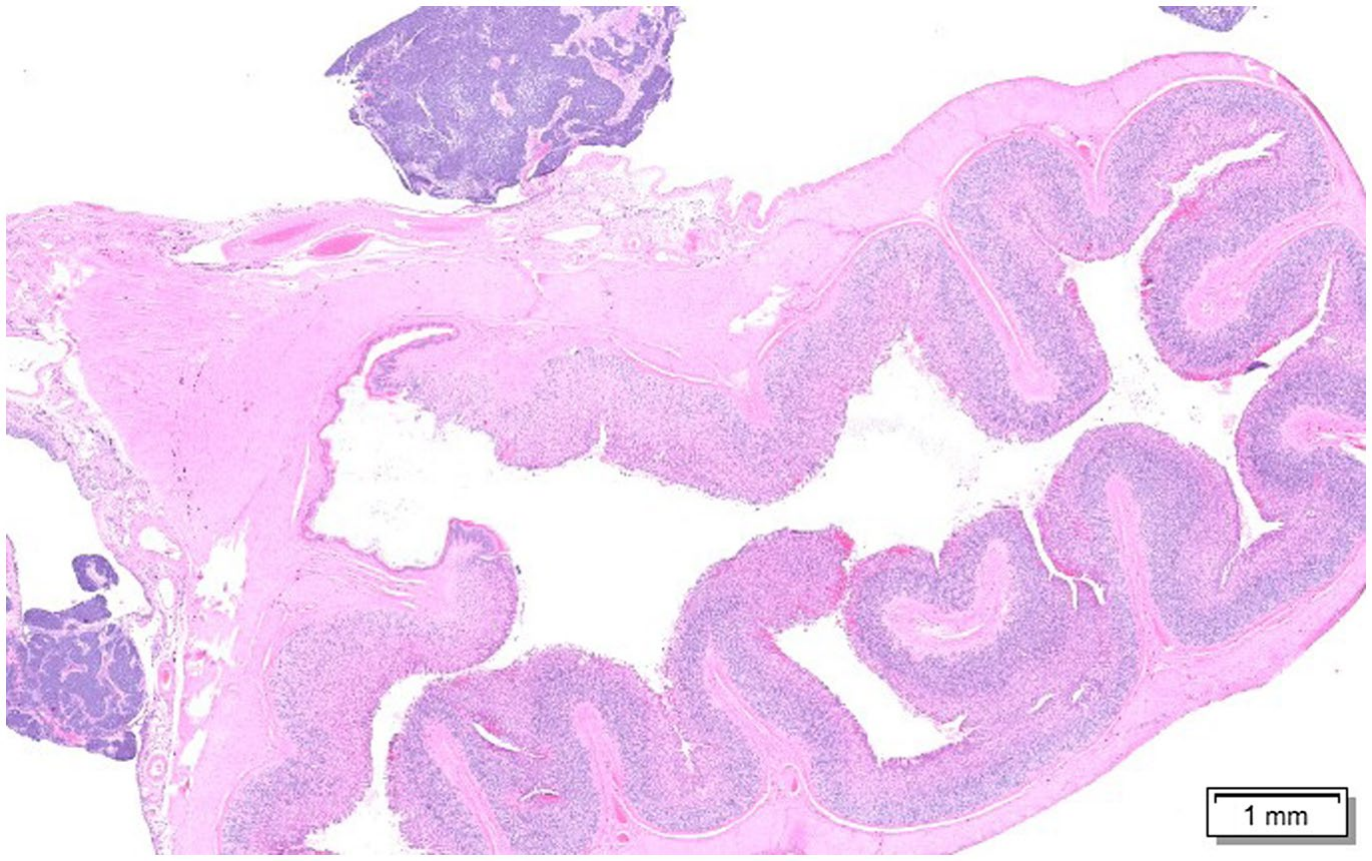
Photomicrograph of the tissue section of the seeding masses attached to the serosa of the stomach (Haematoxylin and eosin [H&E] stain)

Histologically, a neoplastic process was identified in the main abdominal mass which extensively replaced the renal parenchyma. The renal mass was mainly comprised of epithelial (predominant population) and mesenchymal elements (Figure 2). The epithelial elements contained duct‐like structures of monolayer cuboidal epithelium with abundant cytoplasm and elongated oval nuclei (Figure 3). There were one to five mitotic figures detected per one 400× field among blastemal cell population. The mesenchymal element consisted of benign connective tissue formed by loosely arranged spindle cells interspersed with epithelial cells. There were multifocal masses containing similar epithelial cells on the serosal surface of the stomach, pancreas, liver, and intestine (Figure 4). No significant microscopic lesions were detected in the histological sections of the heart and lungs. The case was summarized as a nephroblastoma with peritoneal seeding masses.

## 5 DISCUSSION

Nephroblastoma is an embryonal tumour and represents a distinct and separate pathomorphological entity compared to renal mesenchymal tumour (Alden et al., 1992; Bannasch & Zerban, 1994; Sharma et al., 1994; Yoshizawa et al., 2013). This embryonal tumour originates from the metanephric blastemain humans and animals, but its development in rats has not been fully characterized since embryonal renal tissue is not preserved long after birth (Mesfin, 1999). The usual presentation of nephroblastoma is a unilateral, solitary, well‐demarcated and expansive mass that is sometimes surrounded by a capsule (Alden et al., 1992; Jackson & Kirkpatrick, 2002; Mesfin, 1999; Meuten & Meuten, 2017; Olcott, 1950). This mass is usually a white‐to‐tan, multilobulated, firm mass with occasional spongy and cystic areas (Madheswaran et al., 2009; Mesfin, 1999). The neoplastic mass is located in the cortex, extends through the capsule with focal necrosis and haemorrhage (Chandra et al., 1993; Madheswaran et al., 2009; Meuten & Meuten, 2017). Histologically, nephroblastoma exhibits a triphasic pattern, comprised of blastemal, epithelial and stromal cells (Mesfin, 1999; Meuten & Meuten, 2017; Szychot et al., 2014). Blastemal cells consist of scant and poorly defined basophilic cytoplasm with large nuclei. They are arranged in nests, cords, alveolar, papillary or ductular structures. In the kidney, epithelial differentiation is often intermixed with blastemal cells and comprised of variable combinations and degree of primitive glomeruli and primitive to well‐ differentiated tubules (Alden et al., 1992). Mesenchymal cells form variable amount and combination of areolar tissue to well‐developed fibrous bands. Amongst the blastematous cells and tubular profiles, mitotic activity is relatively frequent (Alden et al., 1992).

In the case presented herein, the pathognomonic features of rat nephroblastoma were recognized and consisted of biphasic population of cells (epithelial and mesenchymal). While Mesfin and Breech have described a rat nephroblastoma comprised of a triphasic morphology (Mesfin & Breech, 1992), most rat nephroblastomas consist of epithelial tumours with the mesenchymal elements being banal non‐malignant stromal tissue (Frazier et al., 2012; Hard & Noble, 1981). In rats, nephroblastomas exhibit a rapid and expansive growth but rarely metastasize (Alden et al., 1992; Bannasch & Zerban, 1994; Chandra & Carlton, 1992; Ito et al., 2014). The uniqueness of this case is the presence of transcoelomic mass due to the expansion of the mass. A similar population of epithelial cells were present in the nodules identified on the serosal surface of liver, pancreas, stomach and small intestine. Rare instances of metastatic spread of neoplastic cells to thoracic cavity (lungs) and abdominal cavity have been recorded (Hard & Grasso, 1976; Katsuta et al., 2017; Mesfin & Breech, 1992; Potkay & Garman, 1969). Interestingly, specific strains of rats are genetically predisposed to this renal tumour (Sprague Dawley subline [Upj:TUC(SD)spf.nb]; Mesfin & Breech, 1992).

One of the main differential diagnoses of nephroblastoma is malignant renal mesenchymal tumour. In rats, ‘renal mesenchymal tumour’ was defined by Hard and Grasso as the predominant mesenchymal tumour observed as a consequence of treatment with chemical carcinogens (Hard & Grasso, 1976). Discrimination of nephroblastoma from this entity is based on the presence of a heterogeneous spectrum of neoplastic spindle cells with an absence of neoplastic epithelial or blastematous component (Alden et al., 1992). However, the early stage of nephroblastoma comprised of focal proliferation of blastematous cells could be mistaken for that entity (Alden et al., 1992). In rats, spontaneous nephroblastoma is uncommon (Bannasch & Zerban, 1994). It is known that nephroblastoma can be induced in the rat by transplacental exposure to direct‐acting alkylating agents such as ethylnitrosourea (Bannasch & Zerban, 1994; Hard, 1985). Infrequently, they may occur following carcinogen exposure of adult rats (Bannasch & Zerban, 1994). The morphological classification of chemically induced nephroblastomas in rats has been questioned in the past due to an undefined distinction between ‘renal mesenchymal tumour’ and nephroblastoma (Bannasch & Zerban, 1994).

The rat in this report was 1 of 10 pups (6 females, 4 males) that were indirectly exposed to titanium dioxide (TiO_2_) while *in utero*. The dam was exposed to TiO_2_ nanomaterial aerosols at an approximate volume of 12 mg/ml per day for 6 non‐consecutive days during mid‐ to late‐gestation. In terms of total maternal lung burden, the estimated amount of TiO_2_ deposited in the lung was between 300–400 µg. Inhalation exposures were during gestational days 12–20. TiO_2_ nanoparticles have been used as a white pigment in paint, a food‐colouring agent, waste‐water remediation agent, and widely used as an ultraviolet blocker in sunscreens. The International Agency for Research on Cancer (IARC) in 2006, classified pigment grade TiO_2_ as a group 2B carcinogen, which may be harmful to humans. Also, the Workplace Hazardous Materials Information System (WHMIS) groups TiO_2_ as D2A, which indicates chemical carcinogenic risk to humans. TiO_2_ nanoparticles (TiO_2_‐NPs) are deposited in the respiratory system through inhalation and then distributed to other organs through vascular circulation. In recent years, studies have shown that after entering the body, TiO_2_‐NPs accumulate in the liver, kidneys, spleen, lungs, heart, and brain. Many studies have also shown that exposure to TiO_2_‐NPs may damage the central nervous system (CNS), but to the author's knowledge there have not been implications of low doses (5 mg/kg) of TiO_2_ causing significant toxicity whereas higher doses (10 mg/kg) caused both lung and liver damage (Relier et al., 2017; Su et al., 2018). No other rats from this litter displayed clinical signs similar to the rat in this case report. Thus, there is no evidence of a relationship between *in utero* exposure to TiO_2_‐NPs and nephroblastomas. This report does not support a nephroblastoma‐induced tumour from indirect exposure of pregnant Sprague Dawley rats by TiO_2_‐NPs.

## CONFLICT OF INTEREST

The authors have no conflict of interest to disclose.

## AUTHOR CONTRIBUTION

**Samantha J Glaspell:** Conceptualization; Data curation; Investigation; Project administration; Writing‐original draft; Writing‐review & editing. **Katie J Knapek:** Data curation; Investigation; Writing‐original draft. **Ida M Washington:** Investigation; Project administration; Supervision; Validation; Writing‐review & editing. **Scott D. Fitzgerald:** Formal analysis; Investigation; Validation; Visualization; Writing‐review & editing. **Jessica S Fortin:** Formal analysis; Investigation; Supervision; Validation; Visualization; Writing‐review & editing.

## ETHICAL STATEMENT

The authors confirm that the ethical policies of the journal, as noted on the journal's author guidelines page, have been adhered to. No ethical approval was required as this is an investigation of an animal at post‐mortem examination.

### Peer Review

The peer review history for this article is available at https://publons.com/publon/10.1002/vms3.405.
